# Paralytic Ileus Secondary to Methamphetamine Abuse: A Rare Case

**DOI:** 10.1155/2017/9762803

**Published:** 2017-08-31

**Authors:** Mark Aidan McKelvie, Yuksel Gercek

**Affiliations:** ^1^School of Clinical Medicine, University of Cambridge, Addenbrooke's Hospital, Cambridge CB2 0SP, UK; ^2^Department of General Surgery, Bedford Hospital NHS Trust, Kempston Road, Bedford MK42 9DJ, UK

## Abstract

Methamphetamine hydrochloride, colloquially referred to as “crystal meth,” is a potent psychoactive amphetamine derivate. Methamphetamine produces stimulant effects in the user including increased energy and alertness as well as inducing intense euphoric symptoms and suppressing appetite through its dopaminergic, serotonergic, and adrenergic actions. Use of methamphetamine can adversely affect cardiovascular, neurological, and gastrointestinal physiology leading to significant morbidity. We present a rare case of paralytic ileus secondary to methamphetamine abuse which has only once before been described in the literature.

## 1. Introduction

Methamphetamine hydrochloride, colloquially referred to as “crystal meth,” is a potent psychoactive amphetamine derivate. Its use produces stimulant effects in the individual user; these include feelings of increased energy and alertness in addition to intense euphoria and appetite suppression [1]. The prevalence of methamphetamine use is lower in the United Kingdom than it is in the United States with a recent Home Office survey revealing 0.2% of 16–24 year olds reporting methamphetamine use in 2014-15 [2]. Comparatively, in the United States, 0.9% of 18–25 year olds had reported use of methamphetamine in 2014-15 [3]. Serious neurologic, cardiovascular, and gastrointestinal harm has previously been reported following methamphetamine use, probably through the downstream effects of monoamine neurotransmitter release [1, 4]. We describe a rare case, hitherto reported only once before in the English-written literature, of methamphetamine-induced paralytic ileus and discuss the possible underlying pathophysiological mechanisms.

## 2. Case Presentation

**Figure 1:**
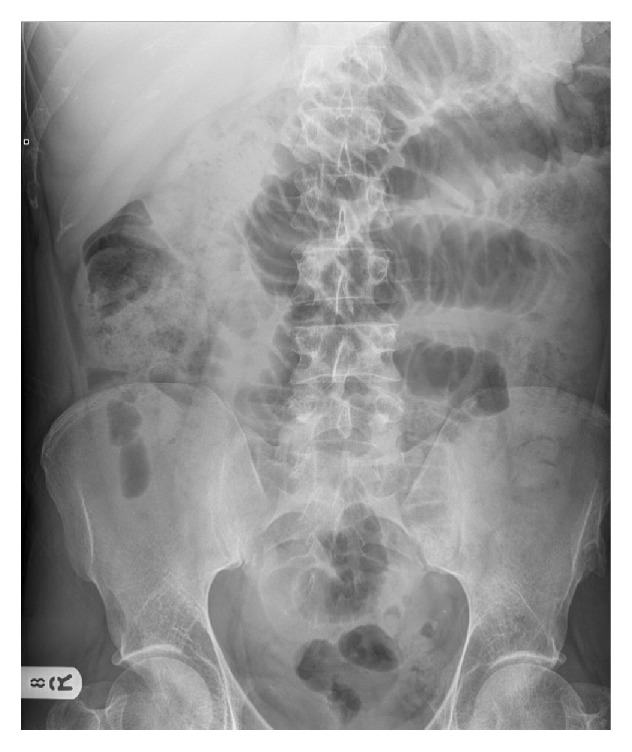
Abdominal plain film radiography demonstrating multiple dilated small bowel loops.

A forty-two-year-old gentleman presented to the emergency department complaining of a one-day history of increasing central abdominal pain and distension. He was nauseated and had vomited watery-brown vomitus five times prior to admission. His bowels had opened the day before but had not opened on the day of admission nor had he passed flatus. His past medical history included regular methamphetamine use (approximately one-quarter ounce, equivalent to approximately seven grams, every five days), previous intravenous drug use, and asthma, for which he regularly used a combined steroid and beta-agonist inhaler. He had had no previous abdominal surgery. He volunteered that in the four days prior to the onset of symptoms he had consumed two to three times his usual amount of methamphetamine, which he swallowed as an oral paste. Physical examination revealed he was mildly dehydrated, with no pallor or icterus. His abdomen was distended and tympanic with absent bowel sounds. There were no signs of peritonism or herniae. Rectal examination revealed a collapsed rectum with no masses. Vital signs showed a tachycardia of 116 beats per minute, blood pressure of 132/84 mmHg, and temperature of 36.0°C. Laboratory studies revealed a white cell count of 14.0 × 10^9^ cells/l (91% neutrophils) and a C-reactive protein of 43 mg/l. Renal and liver function tests, and amylase, were normal and venous gas analysis revealed a pH of 7.39, PaCO2 of 6.0 kPa, serum bicarbonate of 27.2 mmol/l, base excess of 2.2 mmol/l, and serum lactate of 0.9 mmol/l. Dipstick urinalysis was negative and plain film radiography of the chest was unremarkable. Abdominal plain film radiography demonstrated multiple dilated loops of small bowel (Figure 1). The patient was admitted under the general surgical team with paralytic ileus secondary to methamphetamine use, made “nil-by-mouth” and treated with intravenous fluids and a nasogastric tube for gastric decompression. The patient's symptoms began to improve within twenty-four hours of admission after which the nasogastric tube was removed. By forty-eight hours he returned to full oral diet and was discharged home with outpatient drug liaison service follow-up. There had been no recurrence of symptoms since discharge when he was followed up two months later.

## 3. Discussion

Multiple harmful effects, secondary to the use of methamphetamine, on the neurological, cardiovascular, and gastrointestinal systems have been described in the literature. Methamphetamine-induced cerebrovascular accident (both ischaemic and haemorrhagic) [5], neurodegenerative disorders [6], and dyskinesias [7] have been described and cardiovascular complications include acute coronary syndrome [8] and aortic dissection [9]. These effects appear to be related to the stimulated release of neurotransmitters including dopamine, serotonin, and/or noradrenaline [1, 10], with noradrenaline acting via alpha-1 receptors in arterial vasculature to stimulate vasoconstriction and via beta-1 receptors to increase cardiac contractility and heart rate [11], leading to hypertension and tachycardia, and promoting cardiac ischaemia. More recently, a study has demonstrated how methamphetamine directly stimulates the release of endothelin, a potent vasoconstrictor, in mouse arteriole suggesting an additional mechanism in which methamphetamine induces arterial vasoconstriction [12]. With this in mind, methamphetamine-induced ischaemic colitis/infarction has also been described several times before [13, 14], the mechanism of which may also lie in noradrenergic/endothelin-mediated mesenteric arterial vasoconstriction. Carlson et al. [15] have described a single case of paralytic ileus secondary to methamphetamine. Similarly to the case described in our report the patient presented with a short history of abdominal pain, tachycardic, and with abdominal signs consistent with ileus. Plain film radiography in both cases demonstrated multiple dilated bowel loops. Like the patient described in our report, there were no signs of intestinal ischaemia (given the normal venous lactate level). In contrast, the patient described in our report presented with multiple episodes of vomiting and so a decision was made to insert a nasogastric tube to provide gastric decompression and symptomatic relief. The onset of symptoms also clearly coincided with a preceding binge of two to three times the quantity of methamphetamine usually ingested by the patient. With no other obvious aetiology for the ileus, we propose that the cause of the ileus was as a result of the ingestion of a higher-than-usual dose of methamphetamine. Separate to the vascular effects of the monoamine neurotransmitters, there appear to be direct effects of dopamine and noradrenaline on the function of the gastrointestinal tract. Studies implicate dopamine in intestinal motility [16], with one study demonstrating how dopamine, acting at the D1 receptor, causes concentration-dependent reduction in mouse ileum muscle tone and markedly reduces small bowel contractility [17]. Separately, noradrenaline acting on intestinal smooth muscle has been shown to reduce motility [11]. Whilst the exact mechanism for paralytic ileus in the presented case is uncertain, in the absence of evidence of mesenteric ischaemia and other precipitants of ileus, the authors propose that the underlying mechanism for the presentation may involve direct, nonvascular dopaminergic and/or noradrenergic effects on intestinal smooth muscle, secondary to methamphetamine administration, leading to the symptoms and signs of ileus.

The case described is only one of two documented cases of ileus secondary to methamphetamine abuse but reflects a growing trend in recent years of more complications being attributed to the potent stimulant. Whilst these complications are rare, serious sequelae can occur in multiple systems following methamphetamine use, leading to significant morbidity. It is therefore important for the clinician to understand the possible pathophysiological effects of the drug and to consider this as a differential diagnosis in a user of methamphetamine who presents acutely with abdominal symptoms.
